# Current Development and Challenges of Tetravalent Live-Attenuated Dengue Vaccines

**DOI:** 10.3389/fimmu.2022.840104

**Published:** 2022-02-24

**Authors:** Jue Hou, Weijian Ye, Jianzhu Chen

**Affiliations:** ^1^ Antimicrobial Resistance Interdisciplinary Research Group, Singapore-MIT Alliance for Research and Technology (SMART), Singapore, Singapore; ^2^ Department of Biology, Koch Institute for Integrative Cancer Research, Massachusetts Institute of Technology, Cambridge, MA, United States

**Keywords:** live-attenuated vaccine, dengue vaccine, tetravalent vaccine, vaccine development, Dengvaxia^®^

## Abstract

Dengue is the most common arboviral disease caused by one of four distinct but closely related dengue viruses (DENV) and places significant economic and public health burdens in the endemic areas. A dengue vaccine will be important in advancing disease control. However, the effort has been challenged by the requirement to induce effective protection against all four DENV serotypes and the potential adverse effect due to the phenomenon that partial immunity to DENV may worsen the symptoms upon subsequent heterotypic infection. Currently, the most advanced dengue vaccines are all tetravalent and based on recombinant live attenuated viruses. CYD-TDV, developed by Sanofi Pasteur, has been approved but is limited for use in individuals with prior dengue infection. Two other tetravalent live attenuated vaccine candidates: TAK-003 by Takeda and TV003 by National Institute of Allergy and Infectious Diseases, have completed phase 3 and phase 2 clinical trials, respectively. This review focuses on the designs and evaluation of TAK-003 and TV003 vaccine candidates in humans in comparison to the licensed CYD-TDV vaccine. We highlight specific lessons from existing studies and challenges that must be overcome in order to develop a dengue vaccine that confers effective and balanced protection against all four DENV serotypes but with minimal adverse effects.

## Introduction

Dengue is a mosquito-borne viral disease caused by four antigenically distinct serotypes of dengue viruses (DENV1-4). The annual global dengue incidence is about 100 million and is on the rise due to the expansion of mosquito habitat (1). The annual global cost of dengue amounts to about US $8.9 billion in 2016 and is responsible for almost 40000 disability-adjusted life years (2). The dengue viral genome is a positive-sense RNA, which encodes three structural proteins, including the capsid (C), the membrane (prM) and the envelope (E) proteins, and seven nonstructural proteins. The four DENV serotypes share 65-70% nucleotide sequence identity (3). Most dengue infections are either asymptomatic or mild. However, severe dengue in the form of dengue hemorrhagic fever or dengue shock syndrome can occur. Both viral and host factors have been suggested to affect the manifestation of dengue severity (4). For example, the nonstructural protein 1 (NS1) has been shown to act on the vascular endothelium and immune cells, resulting in the release of vasoactive cytokines which cause endothelial hyperpermeability and vascular leakage (5–7).

Following bites by an infected mosquito, DENV initially infects Langerhans cells in the skin. The infection can spread to other phagocytic cells once the infected Langerhans cells migrate to the draining lymph nodes. DENV infection by one serotype usually induces strong homotypic immunity, while cross-reactive heterotypic immunity is usually partial and short-lived (8, 9). This is because cross-reactive antibodies can bind to but do not neutralize heterotypic DENVs due to either low affinity or low titer or both. Instead, such antibody-virus complexes can enhance infection by mediating virus entry into phagocytic cells *via* Fc-gamma receptors in a phenomenon known as antibody-dependent enhancement (ADE), leading to increased risk of severe dengue disease (10). A detailed review on the ADE may be found here (11). Currently, there is no specific antiviral treatment for dengue infection. As such dengue vaccine development is an urgent priority.

An ideal dengue vaccine should confer effective and balanced protection against all four DENV serotypes to minimize ADE. To induce protection against all four DNEV serotypes simultaneously, most dengue vaccines have used tetravalent formulation with the same immunogens, usually prM and E, from each of the four DNEV serotypes. The tetravalent formulation has increased the difficulty in inducing balanced protection due to antigenic competition. Antigenic competition is observed when there is diminution of the immune response to one or more antigen when it is administered together with more immunodominant antigens (12). Consequently, in a tetravalent formulation, it is likely that the immune response will be skewed towards the immunodominant antigen, resulting in biased protective efficacy. Intrinsic differences between the composition of the tetravalent formulation, such as virus replication rate and immunogenicity, can also skew the resulting immune response. Furthermore, ADE has emerged as a significant challenge in dengue vaccine development. Due to the phenomenon of ADE, incomplete protection against all four dengue serotypes can predispose vaccinees to developing more severe dengue if these individuals are subsequently infected by a DENV serotype with suboptimal protection (further discussed under “CYD-TDV Dengue Vaccine”). Indeed, this was observed for CYD-TDV (Dengvaxia), a tetravalent live-attenuated vaccine developed by Sanofi Pasteur (13). Consequently, the Advisory Committee on Immunization Practice (ACIP) only recommends Dengvaxia for children with previous laboratory-confirmed dengue infection (14). In addition, due to the human and non-human primate tropism of DENV, there is a lack of small animal models for DENV-infection and vaccine evaluation. Although mice reconstituted with human immune cells (humanized mice) and mice deficient in type I interferon response have been shown to support DENV infection, there are still limitations to the spectrum of immune responses that can be induced in these mouse models (15, 16).

Currently, there are five types of dengue vaccines under the development, including live attenuated virus vaccines, inactivated virus vaccines, recombinant subunit vaccines, viral vectored vaccines, and DNA vaccines (17). Among the five types of dengue vaccines, the live attenuated virus vaccines are the most advanced as they have been extensively evaluated in humans. Therefore, we focus our review on the live attenuated virus vaccines. The other types of dengue vaccines have been reviewed by Yauch and Shresta (17).

## Live Attenuated Vaccines

Live attenuated vaccines (LAV) use live, but less virulent, pathogens in immunization. As such, they can induce the entire array of antigens required for inducing long-term immune protection (18). LAVs have been successfully developed for other flaviviruses [i.e., yellow fever (YF) and Japanese encephalitis virus (JEV)]. The YF-17D (19) and JEV SA14-14-2 (20) vaccines provide over 90% long-lasting efficacy with a single dose. As natural dengue infection induces life-long homotypic protective immunity, it is expected that LAVs would mimic natural infection and stimulate both cellular and humoral immune responses to confer long-lasting protection. The three most advanced dengue vaccines, CYD-TDV developed by Sanofi Pasteur, TAK-003 developed by Takeda, and TV003/TV005 developed by National Institute of Allergy and Infectious Diseases (NIAID), all use recombinant live attenuated viruses.

## CYD-TDV Dengue Vaccine

**Figure 1:**
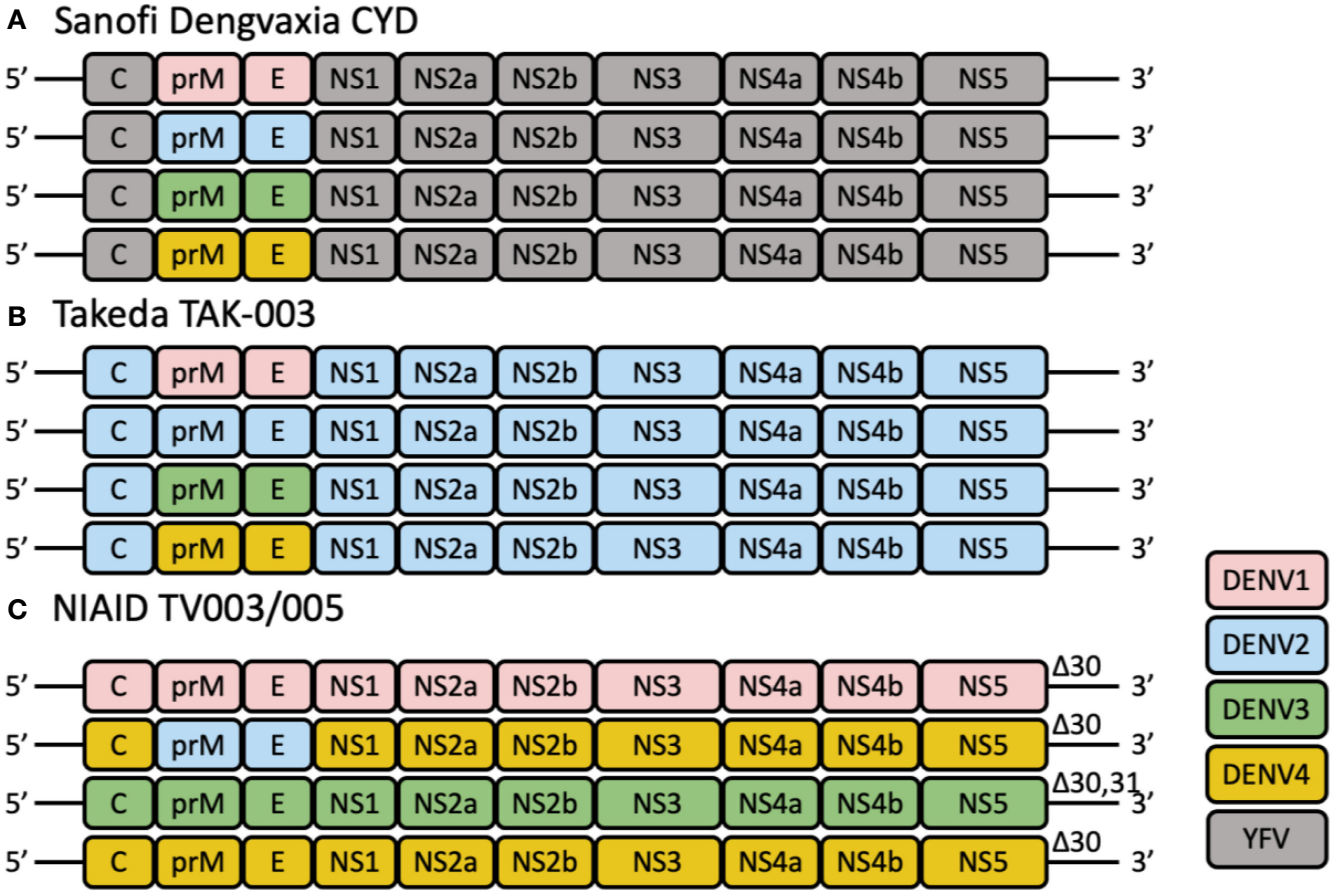
The schematic diagrams of tetravalent live-attenuated dengue vaccines. **(A)** CYD-TDV employs the YFV-17D vaccine strain (shown in gray) as a genetic backbone for the expression of the prM and E genes of DENV1, DENV2, DENV3, and DENV4. **(B)** TAK-003 consists of an attenuated DENV2 strain and three chimeric viruses expressing the prM and E genes of DENV1 (pink), 3 (green) and 4 (yellow) using the DENV2 (blue) genetic backbone. **(C)** TV003/TV005 is composed of three full-length viruses containing all wild-type structural and non-structural genes, and one chimeric virus, in which the prM and E genes of DENV4 are substituted by those of DENV2. These viruses are attenuated by a common 30-nt deletion (Δ30) or 31-nt deletion (Δ31) in the 3′ UTR of the viral genome.

Currently, Sanofi Pasteur’s tetravalent live attenuated CYD-TDV vaccine is the only licensed dengue vaccine. CYD-TDV uses the yellow fever 17D (YF17D) vaccine strain as the backbone and substitutes the YF17D prM and E regions with those of the four DENV serotypes (**Figure 1A**). The overall vaccine efficacy (VE) was in the range of 56.5% to 60.8%. Specific protection against DENV3 and DENV4 were over 70% while protection against DENV1 and DENV2 were 40-50% (21, 22). VE is also affected by the baseline serostatus. As seropositivity rates generally increase with age in an endemic environment, age is often used as a surrogate for DENV exposure (23–25). Though vaccine immunity lasts up to four years (26), the risk of hospitalization for vaccinees was increased 3 years after vaccination (27). Several clinical trials on CYD-TDV in Asia and Latin America had also observed that CYD-TDV was less effective against DENV2 (21, 28, 29). One possible reason for the overall low efficacy is the lack of DENV non-structural (NS) proteins in the formulation. Analysis of the conserved epitopes across the four DENV serotypes revealed that majority of these epitopes are located in the NS proteins (30). The lack of neutralizing antibodies (nAb) and CD8 T cell immune responses against the NS proteins potentially contributes to the reduced protection and durability observed for CYD-TDV (31). However, the imbalanced protection against DENV-2 is not immediately clear as the same 17D backbone was used to express prM and E from each DENV serotype, suggesting either lower expression or lower immunogenicity of prM and E from DENV-2 (see below).

In 2016, CYD-TDV was rolled out in the Philippines and Brazil as part of the national immunization campaign for dengue. However, the immunization campaign was halted in 2017 following the death of 14 children (32). In a study evaluating the long-term safety of CYD-TDV, it was noted that the risk ratio (RR) for dengue-related hospitalization at year 3 follow up was 7.25 (1.15-313.80) for 2-5 years old, 0.63 (0.22–1.83) for 6-11 years old, and 0.25 (0.02–1.74) for 12-14 years old. When participants were divided into those <9 years of age and those >=9, the RR for hospitalization was 1.58 (0.61–4.83) and 0.57 (0.18–1.86), respectively (28). After re-analyzing data from earlier clinical trials, it was concluded that CYD-TDV vaccinated seronegative children between 9-16 years old have a RR of 1.41-1.51 for dengue-related hospitalization and a RR of 1.41-6.25 for severe dengue (27). Such increase in risk of dengue-related hospitalization and severe disease in seronegative participants begin at about 18 months following the last dose of vaccine (27). Further analyses of clinical trial data showed that CYD-TDV vaccination provides the greatest post-vaccination protection against DENV4 and least protection against DENV2. CYD-TDV vaccination further enhances hospitalization risk in seronegative recipients who experienced a later DENV2 infection (33). Consequently, the ACIP recommends the CYD-TDV vaccine to be used for dengue prevention only in children aged 9 to 16 years with prior laboratory-confirmed dengue infection and living in dengue endemic areas (14).

Various hypotheses have been suggested for the increased risk of hospitalization and severe disease in seronegative recipients. As mentioned above, a peculiarity of DENV infection is the phenomenon of ADE due to enhanced entry of antibody-virus complexes into phagocytic cells *via* Fc-gamma receptors. Three types of antibodies – serotype-specific neutralizing antibodies (nAb), cross-reactive antibodies and broad neutralizing antibodies (bnAb), can be induced by either natural DENV infection or through vaccination. Generally serotype-specific nAb are favorable over cross-reactive antibodies, as they bind serotype-specific epitopes and only neutralize the specific DENV serotype (34). Serotype-type specific antibodies have also been shown to be accurate correlates of protection in human dengue infection (35). Cross-reactive antibodies on the other hand, bind but do not neutralize DENV infection (36). Instead, they contribute to the ADE phenomenon by facilitating viral uptake. BnAb recognizes conserved epitopes on all four DENV serotypes and can neutralize all four DENV serotypes. However, these antibodies are rare. One well characterized bnAb targets the E-dimer epitope, and not only neutralizes all four DENV serotype, but also the Zika virus (37). It was shown that CYD-TDV predominantly induced DENV4-specific nAb in seronegative participants, while inducing bnAb and boosting pre-existing DENV type-specific nAb in seropositive participants (38). Consequently, in seronegative individuals, CYD-TDV may induce a DENV4-specific homotypic immune response, thereby mimicking a first infection. As short-lived cross-protective immunity wanes, the more durable anti-DENV4 antibodies thus predispose vaccinees to ADE upon subsequent heterotypic natural DENV infection.

Nonetheless, with the availability of high-resolution dengue transmission maps, even vaccinating only DENV-exposed recipients can potentially reduce global annual dengue incidence by 20-30% (39). However, to improve the adoption and safety of the CYD-TDV vaccine, dengue serostatus diagnostic tests are required (40). Measurement of serum anti-DENV IgG by enzyme-linked immunosorbent assays (ELISAs) is commonly used in laboratories to establish past DENV exposure. However, ELISA are time-consuming and require complex reagents and instruments, as well as staff training (41). Moreover, as a screening test, the specificity of ELISA was observed to be 93.4%, lower than the ideal specificity threshold of 98%. This means that there will be 6.6% of individuals who are falsely seropositive and be at risk of severe dengue if vaccinated (42). Alternatively, rapid dengue diagnostic tests require less handling, are inexpensive, and can be used in the field for quick dengue screening. Nonetheless, these tests remain to be validated in large studies, and may lack sufficient sensitivity and specificity (43).

## TAK-003 Dengue Vaccine Candidate

Takeda’s dengue vaccine (TAK-003) candidate is based on the molecularly characterized live attenuated DENV-2 PDK-53 strain (TDV-2). Three other recombinant chimeric viruses were then engineered using the TDV-2 backbone but with substitution of the DENV-2 prM and E genes with those from DENV-1 16007 (TDV-1), DENV-3 16562 (TDV-3), and DENV-4 1036 (TDV-4) (44). Together, TDV1-4, are formulated as the tetravalent live attenuated dengue vaccine TAK-003 (**Figure 1B**).

DENV-2 PDK-53 was derived from the wild-type DENV-2 16681 virus strain through serial passage in primary dog kidney cells (45). The attenuated DENV2 PDK-53 virus exhibits distinct features that differ from its parental strain, such as temperature sensitivity, less effective replication, attenuated neurovirulence, and greater genetic diversity, which affected the fitness of DENV2 PDK-53 (46). These features are mainly attributed to 5’-NCR-57 C-to-T, NS1-53 Gly-to-Asp, and NS3-250 Glu-to-Val mutations (45). As these mutations lie outside of the structural genes, DENV-2 PDK-53 was used for developing chimeric dengue vaccines that express the structural genes of heterologous dengue viruses while retaining the attenuating phenotypic features.

DENV-2 PDK-53 had been studied as a monovalent or as a single component of the multivalent dengue live-attenuated vaccine candidates in the United States and Thailand. These studies showed that DENV-2 PDK-53 was safe, well-tolerated, highly immunogenic, and generated long-term protection against DENV2 (47–49). The risk of reversal mutations of a live attenuated vaccine is always of concern. Studies suggest that simultaneous back-mutation of two attenuating mutations at 5’-NCR-57 and NS1-53 is sufficient to cause reversion of DENV-2 PDK-53 back to the virulent 16681 wild-type (45). Fortunately, these back-mutations have yet to be observed in the master vaccine seeds of the four serotypes (50).

Preclinical testing revealed that serotype-specific nAb titer elicited by the tetravalent live attenuated vaccine formulation was lower in comparison to the nAb titers elicited through individual monovalent immunization. Of the four DENV components of TAK-003, the chimeric TDV-3 and TDV-4 were less immunogenic and reactogenic than TDV-1 and TDV-2 (44), suggesting viral interference amongst the serotypes. To overcome this issue, several vaccine formulations with differing ratios of the four DENV components were evaluated. Vaccine formulations comprising of an equivalent amount (10^3^ or 10^5^ plaque-forming units, PFU) of each of the four TDV viruses failed to generate adequate nAb against DENV4 after prime and boost immunization in the AG129 mice (51) and cynomolgus monkeys (52). However, the nAb titers against DENV3 and DENV4 were substantially improved in formulations comprising of 10^3^ of TDV-1 and TDV-2 and 10^5^ PFU of TDV-3 and TDV-4. This suggests that the intrinsic differences in the immunogenicity or replication of each DENV serotype in the tetravalent formulations may lead to inter-serotype interference (53). Nonetheless, regardless of formulation composition, the absence of post-challenge viremia in all but one monkey implied that the nAb elicited by the prime-boost vaccination regimes were capable of neutralizing or reducing DENV replication efficiency (52). No adverse clinical events were observed in all the immunized Cynomolgus Macaques (52). Collectively, these preclinical data suggests that formulations comprising of TDV1-4 induce protection against all four DENV serotypes in non-human primates, despite lower antibody responses against DENV4.

### Phase 1 Clinical Trials

Based on preclinical studies and the discrepancies observed in nAb titers against the various DENV serotypes, it appeared that the component ratio of the tetravalent vaccine was critical for the vaccine (51, 52). Two formulations, a low-dose formulation and a high-dose formulation, were assessed in placebo-controlled phase 1 clinical trials (ClinicalTrials.gov NCT01224639, NCT01110551). The low-dose formulation contained 8 × 10^3^, 5 × 10^3^, 1 × 10^4^, and 2 × 10^5^ PFU of TDV1, TDV2, TDV3, and TDV4, respectively, in a ratio of 3.6%: 2.3%: 4.5%: 91.0%, while the high-dose formulation contained 2 × 10^4^, 5 × 10^4^, 1 × 10^5^, and 3 × 10^5^ PFU of TDV1, TDV2, TDV3 and TDV 4, respectively, in a ratio of 4.3%: 10.6%: 21.3%: 63.8% per dose (**Table 1**). Two doses, 90 days apart, were given by subcutaneous or intradermal injections to flavivirus-naïve healthy adults. Regardless of the dose formulation, no clinically meaningful differences in adverse events were observed between vaccine and placebo groups. Majority of adverse events reported included injection site pain and erythema. Overall, no serious adverse events were recorded (54, 55).

**Table 1:** The TAK-003 vaccine compositions of each serotype in the clinical trials.

Phases	Phase1		Phase2			Phase3
Trials	NCT01224639, NCT01110551	NCT01224639, NCT01110551	NCT01511250	NCT02425098	NCT02302066	NCT02747927
TDV1, PFU (% per dose)	8 × 10^3^ (3.59%)	2 × 10^4^ (4.26%)	2 × 10^4^ (4.26%)	2 × 10^4^ (4.71%)	2.5 × 10^4^ (5.40%)	4 × 10^3^ (2.30%)
TDV2, PFU (% per dose)	5 × 10^3^ (2.24%)	5 × 10^4^ (10.64%)	5 × 10^4^ (10.64%)	5 × 10^3^ (1.18%)	6.3 × 10^3^ (1.36%)	1 × 10^4^ (5.75%)
TDV3, PFU (% per dose)	1 × 10^4^ (4.48%)	1 × 10^5^ (21.28%)	1 × 10^5^ (21.28%)	1 × 10^5^ (25.53%)	3.2 × 10^4^ (6.91%)	4 × 10^4^ (22.99%)
TDV4, PFU (% per dose)	2 × 10^5^ (89.69%)	3 × 10^5^ (63.83%)	3 × 10^5^ (63.83%)	3 × 10^5^ (70.59%)	4.0 × 10^5^ (86.34%)	1.2 × 10^5^ (68.97%)
Total PFU of 4 serotypes	2.23 × 10^5^	4.7 × 10^5^	4.7 × 10^5^	4.25 × 10^5^	4.63 × 10^5^	1.74 × 10^5^

Compared to the low-dose formulation, the intradermal high-dose formulation resulted in more than 77.8% seroconversion to DENV1-4 after two doses. Geometric mean titers (GMT) of nAb against DENV1 and DENV2 were also significantly increased in the high-dose formulation. However, in the intradermal high-dose formulation group, although seroconversion to DENV4 was elicited after the second dose, GMT of nAb against DENV4 was the lowest among the serotypes. Nonetheless, regardless of formulation, administration of TDV vaccine-induced detectable neutralizing antibody responses to each of the four serotypes. Overall, GMT to DENV2 was the highest, followed by DENV3, DENV1 and DENV4. Despite the high dose of TDV4 incorporated into the vaccine formulation, DENV4 had the lowest nAb GMT. Regardless of vaccination routes and formulations, the second dose did not substantially increase antibody titers against DENV2, but slightly increased nAb to DENV1, DENV3, and DENV4. This implies that one dose of the vaccine generated sufficient nAb to inhibit or block the replication of DENV2 (54, 55).

### Phase 2 Clinical Trials

The phase 1 trials were implemented in DENV-naïve participants. However, it is important to evaluate the safety and efficacy of dengue vaccines in populations who had been exposed to dengue (DENV-exposed). Therefore, apart from DENV-naïve participants, the high-dose TDV formulation was also evaluated in participants who were seropositive for at least one DENV serotype at baseline (DENV-exposed) in phase 2 clinical trials (ClinicalTrials.gov NCT01511250). Similar to phase 1 trial results, no serious adverse events related to the vaccination were recorded, and the majority of adverse events were injection site pain and erythema (56). In DENV-exposed participants, there was also no increase in adverse events nor vaccine virus replication. As such, it appears that the preexisting antibodies did not increase reactogenicity or the magnitude of virus replication following vaccination (56).

Consistent with the results from the phase 1 studies, the nAb titers to DENV1 and DENV2 were higher than to DENV3 and DENV4 after the first vaccine dose. Moreover, among DENV-naïve participants, more than 94% of participants were seropositive for DENV1, DENV2, or DENV3 on day 28 after the first dose. The second dose increased GMT titers and rates of seropositivity to DENV4 (from 58.6% after the first dose to 87.7% after the second dose). Among the DENV-exposed participants, the rates of seropositivity to any serotype was 91.3% - 99.1%. The second dose slightly increased the rate of seropositivity to DENV1-4 to 96.5% - 100% but had no effect on nAb GMT (56). These results suggest that the TDV is immunogenic regardless of previous dengue exposure.

To improve immune responses TDV-1, TDV-3 and TDV-4, a separate phase 2 clinical trial (ClinicalTrials.gov NCT02425098), where the dose of TDV-2 was reduced by one log unit (5 × 10^3^ PFU), was conducted (57) (**Table 1**). The new formulation elicited a relatively more balanced immune responses with 4-fold lower anti-DENV2 nAb titer compared to previous formulations at day 30, particularly in DENV-naïve subjects. Nonetheless, anti-DENV4 nAb was still suboptimal (57).

Besides optimizing the serotype ratio, the immunization regime and intervals between vaccinations were investigated in a large-scale phase 2 randomized, double-blind, placebo-controlled clinical trial (ClinicalTrials.gov NCT02302066) (58). This trial aimed to determine the safety and immunogenicity of three different vaccination schedules – a two primary dose regime given at 0 and 3 months, a single primary dose regime given at 0 months, a one dose primary regime given at 0 and a booster dose given at 12 months. The vaccine tested comprised of 2.5 × 10^4^ PFU of TDV-1, 6.3 × 10^3^ PFU of TDV-2, 3.2 × 10^4^ PFU of TDV-3, and 4.0 × 10^5^ PFU of TDV-4 (**Table 1**). This formulation had a lower TDV-3 dose compared to the previously tested high-dose formulation, consequently, the ratio of TDV1, TDV2 and TDV4 in the formulation was increased.

Regardless of dosing schedules, the vaccine formulation elicited nAb against all dengue serotypes in vaccinated subjects, with the highest response against DENV2, and lowest response against DENV4 (**Table 2**) (58–60). All serotype-specific nAb were higher compared to baseline based on results published at 6 months (60), 18 months (59) and at 48 months (58), although baseline serostatus impacted nAb titers and seropositivity rates. In DENV-naïve participants, the two primary dose schedules, but not the single primary dose schedule, induced slightly higher long-term persisting nAb titers against DENV1, DENV3, and DENV4, as well as increased tetravalent seropositivity (58–60). Although nAb titers were temporarily increased after the 1-year booster dose, especially in DENV-naïve participants, antibody concentrations and seropositivity rates were similar to the two primary dose schedules by month 48 (58). These results suggest that two-dose schedules, either two primary doses or one primary and one booster, are superior to the one dose schedule in inducing seropositivity rates in participants who are seronegative at the baseline.

**Table 2:** The longitudinal neutralizing antibody responses to four serotype dengue viruses with different dose schedules in a phase 2 trial (NCT02302066).

	Dose schedules	Two-dose primary		One primary		One primary dose plus 1-year boost		placebo	
	Outcomes	Geometric mean titers (95% CI)	Seroconversion rates (%, 95%CI)	Geometric mean titers (95% CI)	Seroconversion rates (%, 95%CI)	Geometric mean titers (95% CI)	Seroconversion rates (%, 95%CI)	Geometric mean titers (95% CI)	Seroconversion rates (%, 95%CI)
6-month	DENV1 GMT	449 (293 to 688)	100.0 (95.7 to 100.0)	464 (330 to 653)	97.1 (93.3 to 99.0)	539 (391 to 742)	99.4 (96.8 to 100.0)	56 (29 to 106)	49.4 (38.1 to 60.7)
	DENV2 GMT	1462(1072 to 1993)	98.8 (93.5 to 100.0)	1683 (1334 to 2124)	99.4 (96.8 to 100.0)	1335 (1067 to 1670)	99.4 (96.8 to 100.0)	77 (40 to 150)	49.4 (38.1 to 60.7)
	DENV3 GMT	150 (97 to 233)	98.8 (93.5 to 100.0)	166 (118 to 235)	92.9 (88.0 to 96.3)	174 (126 to 241)	93.1 (88.3 to 96.4)	37 (22 to 65)	48.1 (36.9 to 59.5)
	DENV4 GMT	109 (73 to 163)	92.8 (84.9 to 97.3)	110 (81 to 149)	90.6 (85.2 to 94.5)	92 (69 to 124)	85.1 (78.9 to 90.0)	22 (14 to 35)	44.4 (33.4 to 55.9)
18-month	DENV1 GMT	476 (286 to 791)	95.1 (87.8 to 98.6)	461 (329 to 647)	97.0 (93.2 to 99.0)	1056 (804 to 1388)	98.8 (95.9 to 99.9)	92 (49 to 173)	62.5 (51.0 to 73.1)
	DENV2 GMT	1212 (842 to 1744)	98.8 (93.3 to 100.0)	1242 (947 to 1628)	97.6 (94.0 to 99.3)	1457 (1182 to 1796)	100.0 (97.9 to 100.0)	177 (93 to 337)	68.8 (57.4 to 78.7)
	DENV3 GMT	286 (171 to 478)	95.1 (87.8 to 98.6)	298 (205 to 433)	92.3 (87.1 to 95.8)	548 (411 to 730)	98.3 (95.0 to 99.6)	78 (44 to 137)	63.8 (52.2 to 74.2)
	DENV4 GMT	98 (65 to 150)	87.7 (78.5 to 93.9)	102 (75 to 139)	86.9 (80.8 to 91.6)	172 (133 to 222)	97.1 (93.3 to 99.0)	33 (21 to 52)	57.5 (45.9 to 68.5)
48-month	DENV1 GMT	378 (226 to 632)	96.9 (89.3 to 99.6)	421 (285 to 622)	94.7 (89.5 to 97.9)	719 (538 to 960)	100.0 (97.3 to 100.0)	100 (50 to 201)	68.3 (55.3 to 79.4)
	DENV2 GMT	1052 (732 to 1511)	100.0 (94.5 to 100.0)	1319 (970 to 1794)	98.5 (94.7 to 99.8)	1200 (927 to 1553)	100.0 (97.3 to 100.0)	208 (99 to 437)	68.3 (55.3 to 79.4)
	DENV3 GMT	183 (113 to 298)	95.4 (87.1 to 99.0)	201 (135 to 298)	90.2 (83.9 to 94.7)	288 (211 to 392)	97.8 (93.7 to 99.5)	71 (37 to 139)	63.5 (50.4 to 75.3)
	DENV4 GMT	152 (97 to 239)	90.8 (81.0 to 96.5)	164 (114 to 236)	91.0 (84.8 to 95.3)	219 (165 to 290)	99.3 (96.0 to 100.0)	46 (26 to 82)	60.3 (47.2 to 72.4)

### Phase 3 Clinical Trials

The efficacy of TAK-003 was evaluated in a phase3 double-blind, randomized, placebo-controlled trial (ClinicalTrials.gov NCT02747927) (61, 62). This trial involved healthy children and adolescents between the ages of 4 to 16 years in eight dengue-endemic countries. Two doses of vaccine or placebo were administered 3 months apart. Each dose of TAK-003 contained 4 × 10^3^ (3.6 log10), 1 × 10^4^ (4.0 log10), 4 × 10^4^ (4.6 log10), and 1.2 × 10^5^ (5.1 log10) PFU of TDV-1, TDV-2, TDV-3, and TDV-4, respectively (**Table 1**).

At the end of 12 months follow-up post-second vaccination, the overall VE in the per-protocol population was 80.9% (95% CI, 75.2 to 85.3), with 95.4% efficacy against dengue-related hospitalization (95% CI, 88.4 to 98.2) (62). The efficacy varied among the serotypes, with reported efficacies of 73.7%, 97.7%, 62.6% against DENV1, DENV2, and DENV3 respectively (62). The results for efficacy against DENV-4 were inconclusive due to the low number of DENV-4 cases (61, 62). VE was similar across age ranges (72.8% to 83.3%). In participants who were seronegative at baseline, VE was 74.9%, and in those who were seropositive at baseline, VE was 82.2%. Against DENV1 and DENV2, VE was 79.8% and 96.5% respectively, among DENV-exposed participants; and was 67.2% and 100% respectively, among DENV-naïve participants. Due to the limited number of confirmed dengue cases caused by DENV3 and DENV4, the results for DENV3 and DENV4 are inconclusive. Though inconclusive, no efficacy was suggested against DENV3 (62).

At the end of 18 months follow-up post-second vaccination, a cumulative VE of 80.2% (95% CI 73.3 to 85.3) was reported (61). Analysis of secondary assessment timeframe (i.e., between 12-18 months) showed that overall VE was 73.3% (95% CI 66·5 to 78·8). VE was 76.1% (95% CI 68.5 to 81.9) in DENV-exposed individuals, and 66.2% (49.1 to 77.5) in DENV-naïve individuals. Against dengue-related hospitalization and dengue hemorrhagic fever, VE was 90.4% (82.6 to 94.7) and 85.9% (31.9 to 97.1), respectively. Efficacy also varied among the DENV serotypes: 69.8% (95% CI 54.8 to 79.9 for DENV1, 95.1% (89.9 to 97.6) for DENV2, 48.9% (27.2 to 64.1) for DENV3, and 51.0% (–69.4 to 85.8) for DENV4. Notably, no VE was shown in seronegative in DENV-naïve individuals against DENV3. Moreover, though statistically inconclusive, TAK-003 led to more hospitalization in DENV-naïve individuals because of DENV3 compared to placebo (61). Data released at the end of 24 months follow-up after vaccination showed that cumulative overall VE was 72.7% (95% CI 67.1 to 77.3). In DENV-exposed individuals VE was 74.8% (95% CI, 68.6%-79.8%), and 67% (95% CI, 53.6%-76.5%) in DENV-naïve participants. Cumulative serotype-specific efficacy against DENV1, DENV2, and DENV3 were 69.0%, 90.8%, and 51.4% respectively. Efficacy against DENV4 was inconclusive. However, analysis of VE in the second year showed that VE had declined to 56.2% (95% CI, 42.3%-66.8%). This drop in VE was partially attributed to a change in annual serotype dominance variation (63). Lack of VE against DENV3 in DENV-naïve individuals persisted into the second year (63).

More recently, 36 months follow-up data was released (64). TAK-003 demonstrated cumulative overall VE of 62.0% (95% CI: 56.6% to 66.7%) against virologically confirmed dengue. Amongst DENV-exposed individuals, VE was 65.0% (95% CI: 58.9% to 70.1%) and among DENV-naïve individuals, VE was 54.3% VE (95% CI: 41.9% to 64.1%). These results suggest that there was a gradual waning of VE from 12 months to 36 months post-immunization. Similar to earlier results, no VE was observed against DENV3 in DENV-naïve individuals. TAK-003 also led to a higher hospitalization rate in DENV-naïve individuals (0.2%) because of DENV3 compared to placebo (<0.1%). Such a phenomenon should be followed up in future studies as it could be an early signal of ADE in seronegative recipients when exposed to DENV3 (65).

## Comparison of CYD-TDV and TAK-003 Vaccine Efficacies to DENV Serotypes

Both CYD-TDV and TAK-003 vaccines are tetravalent live-attenuated viruses. In CYD-TDV, the prM and E proteins from each DENV serotype are expressed from the YF17D backbone and formulated into the same tetravalent vaccine. However, protection efficacy was significantly different against different DENV serotypes: highest against DENV3 and DENV4 and lowest against DENV2. Similarly, in TAK-003, the prM and E proteins from each DENV serotype are expressed from the DENV2 backbone and formulated into the same tetravalent vaccine. However, protection efficacy was highest against DENV2 and lowest against DENV4. Multiple factors likely contribute to the observed differences in protection efficacy with the two vaccines. The results appear to suggest different immunogenicities of the serotype-specific DENV components (prM and E) in the two vaccine formulations. This could be due to intrinsic differences in immunogenicities of prM and E from different DENV serotypes and/or differences in infectivity and replication of the four chimeric viruses. Antigenic interference or competition among the four chimeric viruses in the tetravalent vaccines, especially when the same amount of each chimeric virus was used, could also affect the balanced generation of nAb against all four serotypes. Despite Takeda’s attempt in adjusting formulation component ratios to elicit a more balanced response, the improvement observed was limited. For example, despite the changes in vaccine composition throughout phase 2 and phase 3 trials, the geometric mean titer of nAb was still imbalanced, with anti-DENV4 the lowest and anti-DENV2 the highest. Notably, protection efficacy against DENV2 is lowest for CYD-TDV but highest for TAK-003. This result would suggest that prM and E from DENV2 are not intrinsically less immunogenic. However, another difference between the two vaccines is that TAK-003 contains not only prM and E from DENV2 but also capsid protein and all seven nonstructural proteins from DENV2, in contrast, CYD-TDV contains only prM and E. Immune responses to capsid and nonstructural proteins in TAK-003 likely contributed to the high protection efficacy against DENV2 (see discussion on CD8^+^ T cell responses below).

## TV003/TV005 Dengue Vaccine Candidate

In contrast to utilizing a single common backbone vector in CYD-TDV and TAK-003 formulations, the NIAID investigators took a different approach in developing tetravalent live attenuated dengue vaccines. They created a series of attenuated DENV by introducing nucleotide deletions in the 3’ untranslated region (UTR) and additional mutations in nonstructural proteins (66). Six monovalent DENV vaccine candidates were evaluated in mouse and non-human primates and finally four of them that covering all four serotypes were selected for inclusion into a tetravalent vaccine formulation (67). These monovalent DENV vaccine candidates are as follows: rDEN1Δ30 (68), rDEN2/4Δ30 (69, 70), rDEN3-3′D4Δ30 (71), rDEN3Δ30/31 (71), rDEN4Δ30 (72), and rDEN4Δ30-200,201 (73, 74). rDEN1Δ30 and rDEN4Δ30 were generated through the introduction of a 30-nucleotide deletion (Δ30) into the 3ʹUTR of DENV1 and DENV4 genomes, respectively. In addition to the Δ30 deletion, rDEN3Δ30/31 includes an additional 31 nucleotide deletion located 55 nucleotides upstream of the Δ30 mutation. rDEN2/4Δ30 was a chimeric virus created by substituting the prM and E gene segments of rDEN4Δ30 with those derived from DENV2. Similarly, rDEN3-3′D4Δ30 was a chimeric virus created by replacing the entire 3′UTR of DENV3 with the 3′ UTR of rDEN4Δ30. rDEN4Δ30-200,201 is based on its rDEN4Δ30 parent and contained alanine substitutions at amino acid position 200 and 201 of the NS5 proteins. Prior studies also showed that these attenuated viruses were incapable of being transmitted by mosquitoes (75).

### Phase 1 Clinical Trials

Four different tetravalent admixtures (TV001-004) of the six monovalent LAV were evaluated in flavivirus-naïve adults in a randomized, double-blind phase 1 clinical trial (ClinicalTrials.gov NCT01072786) (**Table 3**) (67). Regardless of the admixture tested, no difference was observed in the incidence of adverse events between participants who received vaccines or placebos. Majority of the adverse events involved mild rash (76). Admixture TV003 consisting of rDEN1Δ30, rDEN2/4Δ30, rDEN3Δ30/31, and rDEN4Δ30, appeared to induce the most balanced antibody responses across the four DENV serotypes (**Figure 1C**). 97% of vaccinees developed trivalent nAb responses after receiving a single dose of TV003. In participants who were flavivirus-experienced prior to vaccination, TV003 induced slightly higher DENV3 viremia, higher neutralizing antibody titers to DENV2, -3, and -4, and a higher tetravalent response frequency (77). It was demonstrated in a human DENV2 challenge model that TV003 elicited complete protection against dengue (76).

**Table 3:** The NIAID vaccine candidate composition of each serotype.

Admixture	Administered dose of each component (log10 PFU)	DENV1	DENV2	DENV3	DENV4
TV001	3, 3, 3, 3	rDEN1Δ30	rDEN2/4Δ30	rDEN3-3′D4Δ30	rDEN4Δ30
TV002	3, 3, 3, 3	rDEN1Δ30	rDEN2/4Δ30	rDEN3-3′D4Δ30	rDEN4Δ30-200,201
TV003	3, 3, 3, 3	rDEN1Δ30	rDEN2/4Δ30	rDEN3Δ30/31	rDEN4Δ30
TV004	3, 3, 3, 3	rDEN1Δ30	rDEN2/4Δ30	rDEN3Δ30/31	rDEN4Δ30-200,201
TV005	3, 4, 3, 3	rDEN1Δ30	rDEN2/4Δ30	rDEN3Δ30/31	rDEN4Δ30

A booster dose of TV003 administered either at 6 months (ClinicalTrials.gov NCT01072786) or 12 months (ClinicalTrials.gov NCT01782300) after the primary dose was also evaluated in flavivirus-naïve participants to determine if a booster dose can improve nAb titers (78, 79). No major adverse events related to vaccination were observed for both trials. For the 12 months booster trial, the only statistically significant adverse event reported by 63% of vaccinees was a mild vaccine-associated rash which lasted on average 7.7 days (79). Although a booster TV003 dose administered at 6 months increased seroconversion rates to DENV2 from 76% to 94%, a second TV003 dose was not found to significantly increase mean peak nAb titers to any serotype (78). Similarly, a booster TV003 dose administered at 12 months did not significantly boost nAb titers to any serotype (79). Additionally, the first vaccine dose induced sterilizing immunity capable of neutralizing the booster vaccine dose. As such, a booster dose of TV003 was unnecessary and provided only minimal benefits.

To increase the immune response towards DENV2, DENV2 dose was increased by 10-fold in TV005 (**Table 3**). TV005 was then evaluated in two clinical trials (ClinicalTrials.gov NCT01072786 and NCT01436422) (78). In both trials, no TV005-related serious adverse events were noted, and the occurrence of adverse events were not significantly different between TV003 and TV005. TV005 significantly improves seroconversion frequencies and overall antibody titers to DENV2 while maintaining the immunogenicity of the other serotypes. A single dose of TV005 was shown to elicit a tetravalent response in 90% of vaccinees, compared to 76% for TV003, by the third month after vaccination.

### Phase 2 Clinical Trial

The Butantan Institute licensed TV003 from NIAID and manufactured Butantan-DV, which is analogous to, but not the same as, TV003. Subsequently, Butantan-DV was evaluated in a phase 2 clinical trial (ClinicalTrials.gov NCT01696422) (80). The trial recruited both DENV-naïve and DENV-exposed participants. A dosing regimen involving a single dose of Butantan-DV followed by a booster dose given at six months apart was investigated. Similar to prior results, self-limiting rash was the most common adverse event that occurred in the vaccinated group (88%-92%) compared to placebo group. No significant differences in the frequency of unsolicited adverse reactions were observed between DENV-naïve and DENV-exposed participants. No significant differences in viremia post-vaccination between DENV-naive and DENV-exposed participants were observed. 91 days after the first dose, the overall seroconversion rates to DENV1, DENV2, DENV3 and DENV4 were 94%, 82%, 82% and 88% respectively. Seroconversions rates were significantly higher for DENV2 (92% vs 78%) and DENV4 (89% vs 77%) in DENV-naïve participants compared to DENV-exposed participants. No significant differences in seroconversion frequency were observed between DENV-naïve and DENV-exposed participants for DENV1 (87% vs 81%) and DENV3 (76% vs 82%). However, GMT of nAb were significantly higher in DENV-exposed participants for DENV1, DENV2 and DENV3, but not DENV4. Following the booster dose, nAb GMT and seroconversion rates were not significantly improved. This corroborates with earlier observations that a single TV003 dose was sufficient to elicit protective immunity against dengue (78).

TV005 is currently undergoing phase 2 clinical trial in Taiwan (ClinicalTrials.gov NCT04133987), while a phase 3 clinical trial for Butantan-DV had been registered at ClinicalTrials.gov (NCT02406729).

## Cellular Immune Responses Post-Vaccinations

Generating an effective cellular immune memory is a hallmark of LAV and the importance of cellular immune memory has been validated in various vaccine studies (81–83). The cellular immune responses after natural dengue infections mainly involved recognition of NS1, NS2A and NS3 (84–86), which were absent in the CYD-TDV vaccine. Instead, the CYD-TDV vaccine elicited mostly YF-17D NS3 specific CD8^+^ T cell responses and DENV serotype-specific CD4^+^ T cell responses (87). By using DENV2 PDK-53 as a backbone vector, the TAK-003 vaccine promoted significant CD8^+^ T cell activation and moderate CD4^+^ T cell activation. The elicited cellular immune responses persisted for at least 120 days after vaccination and its specificity span the DENV proteome with preference for NS1, NS3, and NS5 peptides (88). Since nonstructural proteins in TAK-003 are all from DENV2, the total cellular responses to DENV2 NS proteins far exceeded the responses elicited from the other three DENV serotypes (88). Collectively, these studies suggest that when YFV or DENV2 was used as the sole vaccine backbone, cellular immune responses were restricted to backbone-dominated T cell responses.

By incorporating the NS proteins from three DENV serotypes (DENV1, DENV3 and DENV4), the TV003 vaccine induced broad and cross-reactive T cell responses. Each component of the TV003 tetravalent vaccine elicited detectable CD8^+^ T cell responses comparable to natural dengue infection. ELISPOT assays performed on PBMCs from monovalent vaccinated participants showed that structural proteins and NS proteins were responsible for 10-40% and 60-90% of the total IFN-γ responses, respectively (89). Strikingly, in the PBMCs of participants vaccinated with the tetravalent TV003 vaccine, NS proteins accounted for 99.8% of IFN-γ responses elicited, and highly conserved epitopes in NS3 and NS5 were responsible for 93% of the responses (89). Data from the TV003 phase 2 clinical trial also showed that 91 days after the first TV003 dose, 94% of participants had antigen-specific CD8^+^ T cell IFN-γ response. These CD8^+^ T cell responses were not significantly different between DENV-naïve and DENV-exposed participants (80).

In contrast to the CD8^+^ responses induced by NS3 and NS5 proteins, CD4^+^ T cells induced by TV005 vaccination dominantly recognized capsid, NS2A and NS5 proteins, thereby suggesting differences in immunodominance pattern in the NS proteins. Such immunodominance observed after TV005 vaccination was similar to those observed in natural infection (90).

## Lessons and Challenges of Dengue Vaccine Development

One of the most important lessons in dengue vaccine development, is the occurrence of breakthrough dengue infection following DENV vaccination. Given the dual roles that antibodies play in controlling DENV infection, it is important that vaccines stimulate a balanced nAb response against all four DENV serotypes to achieve optimal protection with no or minimal ADE. This is especially important in seronegative vaccinees, where partially protective DENV vaccines can mimic a first dengue infection. As short-term vaccine-induced heterotypic protection decline, the vaccinee is left with suboptimal homotypic (i.e., from an immunodominant serotype) protection, which can predispose them to severe dengue when a breakthrough infection occurs. Thus, during vaccine development, baseline serostatus of vaccinees, serotype-specific differences in efficacy, and durability of protection should always be considered (91).

Second, it is critical to consider the targets of nAb when designing dengue vaccines. For example, studies have shown that highly specific and neutralizing antibodies primarily target the EDIII domain of the E protein, while weak cross-reactive antibodies target prM (92, 93). As such detailed characterization of the epitopes of vaccine-induced antibodies is important. Antibody depletion studies in TV003 vaccinees showed that 62%, 76%, 86% and 100% of vaccinees developed type-specific (TS) nAb to DENV1, DENV2, DENV3 and DENV4, respectively. 48% of vaccinees had TS nAb to all four DENV serotype, while another 29% of vaccinees had TS nAb to 3 out of 4 DENV serotypes. These TS nAb generally map to epitopes on domains on the E protein (94). In contrast, 5%, 83%, 12% and 27% of TAK-003 vaccinees developed TS nAb to DENV1, DENV2, DENV3 and DENV4, respectively. Most of the anti-DENV2 TS nAb bind to DENV2 EDIII epitopes (95). To avoid the generation of anti-prM, a tetravalent subunit virus-like particle vaccine, DSV4, which expresses EDIII of all four DENVs but not prM, had been engineered. In mouse models, DSV4 was shown to elicit mainly type-specific antibodies and protected mice from lethal DENV challenge without promoting ADE (96).

Third, given the importance of generating broad nAb (bnAb), alternative approaches to vaccine design can be considered. Different strategies had been developed to identify such bnAb. Hu et al. employed a competitive sorting strategy utilizing yeast surface display of a naïve single chain antibody library isolated from human donors and identified a bnAb targeting domain III of the E protein (97). Another strategy involved characterizing B cells isolated from DENV-vaccinated macaques. Six bnAbs targeting various epitopes on the E protein were identified (98). A third strategy involving characterizing plasmablasts from DENV-infected patients led to the identification of two bnAb targeting domain I of the E protein (99). However, it remains to be seen if bnAb identified in these *in vitro* screens can translate to *in vivo* neutralizing activity. Indeed, Durham et al. noted that the two bnAbs they identified contributed minimally to the overall neutralizing activity of the patients’ serum (99). Another challenge is how an immunogen that triggers these bnAbs can be reverse engineered to become a vaccine candidate.

Fourth, given the importance of neutralizing antibodies in conferring protection against DENV infection, a reliable test to measure such immune responses is critical. The plaque reduction neutralization test (PRNT) is commonly used to determine DENV neutralizing antibodies and immunogenicity of vaccine candidates. Although the WHO has released its guidelines on the performance of the PRNT assay (100), the PRNT is still highly variable depending on assay reagents and conditions (101). For example, it was reported that inter-laboratory differences account for about 50% of variations in PRNT titers (102). Also, the correlation between neutralizing antibodies and DENV-infection protection is not absolute (103–107). As such, there is a pressing need to identify and develop assays to better identify surrogates of DENV protection.

Fifth, clinically relevant immune mechanisms change over time following natural infection or vaccination. For example, high nAb titers might be important in conferring immunity immediately post-vaccination, but a good CD4 response might be required for longer term maintenance of B cell memory (108). Consequently, the inclusion of T cell epitopes in the vaccines may increase vaccine efficacy. Most of the CD8 and CD4 T cell epitopes are found in the non-structural proteins (i.e. NS2A/B NS3, and NS5), and the presence of these T cell epitopes in TV003 have been suggested to partially contribute to its observed efficacy with a single dose (109). Alternatively, strategies such as sequential monovalent heterologous immunizations have also been shown to promote T cell responses post-vaccination in mice (110, 111).

Sixth, given the similarities in the structure of the E protein among flaviviruses, such as yellow fever virus (YFV), Zika virus and Japanese encephalitis virus (JEV), there is a possibility that prior flavivirus infection may generate cross-reactive DENV antibodies, resulting in ADE. For example, it was suggested that pre-existing anti-JEV antibodies can increase YFV viremia after YFV vaccination (112). Pre-existing DENV antibodies were also noted to either protect against Zika infection or potentiate Zika infection through ADE, depending on antibody concentration (113). In mouse models, CYD-TDV enhances Zika virus infection through ADE (96). Thus, it is important to evaluate the risk of ADE from vaccination in flavivirus-exposed population. Furthermore, considering the increased risk of dengue-related hospitalization and severe dengue manifest between 18 months and 3 years after the last dose of vaccine, a longer post-vaccination surveillance is required to assess the overall safety of dengue vaccines.

In summary, development of a dengue vaccine that confers effective protection against all four DENV serotypes with no or minimal of ADE remains a challenge. The different designs of tetravalent live attenuated dengue vaccines and their extensive evaluations in humans so far have shed light on the vaccine design considerations. With infusion of new technologies, such as mRNA-based vaccines, an effective dengue vaccine should be within the reach in the near future.

## Author Contributions

JH and WY drafted this manuscript and JH, WY, and JC revised the manuscript. All authors contributed to the article and approved the submitted version.

## Funding

This work was supported by the National Research Foundation of Singapore through the Singapore–MIT Alliance for Research and Technology’s Interdisciplinary Research Group in Antimicrobial Resistance Research Program.

## Conflict of Interest

The authors declare that the research was conducted in the absence of any commercial or financial relationships that could be construed as a potential conflict of interest.

## Publisher’s Note

All claims expressed in this article are solely those of the authors and do not necessarily represent those of their affiliated organizations, or those of the publisher, the editors and the reviewers. Any product that may be evaluated in this article, or claim that may be made by its manufacturer, is not guaranteed or endorsed by the publisher.
